# Factors Related to Intentions to Commit Dating Violence among Taiwanese University Students: Application of the Extended Theory of Planned Behavior

**DOI:** 10.3390/ijerph18041956

**Published:** 2021-02-17

**Authors:** Chung-Ying Lin, Ying-Hua Tseng, Mei-Ling Lin, Wen-Li Hou

**Affiliations:** 1 Institute of Allied Health Sciences, College of Medicine, National Cheng Kung University, Tainan 70101, Taiwan; cylin36933@gmail.com; 2 Department of Public Health, National Cheng Kung University Hospital, College of Medicine, National Cheng Kung University, Tainan 70101, Taiwan; 3 Department of Occupational Therapy, College of Medicine, National Cheng Kung University, Tainan 70101, Taiwan; 4 School of Nursing, College of Nursing, Kaohsiung Medical University, Kaohsiung 80708, Taiwan; yhtseng@kmu.edu.tw; 5 Department of Nursing, College of Nursing, Hungkuang University, Taichung 43304, Taiwan; linml@sunrise.hk.edu.tw; 6 Department of Medical Research, Kaohsiung Medical University Hospital, Kaohsiung 80708, Taiwan

**Keywords:** behavioral intention, dating violence, theory of planned behavior (TPB), gender stereotyping, history of family violence

## Abstract

Dating violence (DV) constitutes a major public health and safety issue worldwide; however, only limited research into this important subject has been conducted in Taiwan. This study examined university students’ intention to commit DV, based on the expanded theory of planned behavior (TPB), with a history of family violence and gender stereotyping also included as further factors in the original TPB model. A total random sample consisting of 450 university students from four universities in four regions in Taiwan, namely, the northern, southern, central, and eastern regions, participated. Of these participants, 365 (81.1%) completed all of the parts of the questionnaires, which included a survey of demographic data, such as any history of family violence; a gender stereotyping questionnaire; and a DV behavioral intention questionnaire. The results showed that the three main variables of the TPB—that is, subjective norms, attitudes, and perceived behavioral control—significantly related to university students’ intentions to commit DV. More specifically, university students’ attitudes and subjective norms emerged as significant related factors of their intention to commit DV behaviors. Overall, the expanded TPB explained 30.4% of the variance in DV intentions, and attitude was the most significant factors after controlling the background variables. These findings can hopefully be used to help design and implement programs for the prevention of DV behaviors among university students.

## 1. Introduction

Dating violence (DV) is a significant public health and safety problem in Taiwan [1] and around the world [2,3]. Youths between the ages of 16 and 24 are most likely at a period in their lifecycle to develop a dating relationship and were in their most dangerous period for intimate partner violence [4]. An early DV experience may cause acute and chronic physical and mental health problems [5,6,7] and may cause someone to become a perpetrator or a victim of domestic violence [8]. In America, 66% of college students have experienced DV as at least one physical, sexual, or verbal assault [9]. In Taiwan, almost 60% of college students have suffered from DV [10,11]. The Ministry of Health and Welfare in Taiwan (2017) [1] reported that the number of perpetrators (ages 18–24) notified of domestic violence incidents had gradually increased from 3050 in 2015 to 4782 in 2019. Moreover, school DV incidents have become more frequent, and “terrorist lovers’” self-injury and homicides are worrisome and distressing in recent years.

In response, the Taiwan government has amended the Domestic Violence Prevention Law to include those over 16 years of age who were physically or mentally infringed upon by an intimate partner while not being in a common-law relationship. They have been included and protected by Taiwan’s Domestic Violence Prevention Law since 4 February 2016. The above information shows that DV is an important issue among youths in Taiwan. College students are the population best known for developing intimate romantic relationships more frequently than other groups [12]. Hence, we need to better understand the decision-making process about committing DV to reduce the incidences of this violent behavior among university students.

Formulated as a means of predicting and explaining human behaviors, the theory of planned behavior (TPB) [13] has been widely utilized in social psychology and social psychology-related research [14,15]. According to the TPB, a “behavioral intention” indicates how willing an individual is to engage in a particular behavior, such that it likewise constitutes the most accurate factor of that individual’s actual behavior. The TPB further holds that a behavioral intention is preceded by the following three cognitive antecedents: (1) subjective norms, which consist of perceptions regarding the social pressure to engage or not engage in the given behavior; (2) attitudes, which refer to the individual’s own positive or negative views regarding the behavior; and (3) perceived behavioral control, which refers to the perceived difficulty or ease of performing a behavior (in other words, the individual’s confidence or lack thereof that the individual can perform the behavior), with this antecedent also regarded as being directly predictive of behavior. These three antecedents have further been described as internal psychological factors that effectively predict the behavioral intentions of an individual, and they are further regarded as being changeable.

To date, only a few studies using the TPB [16,17] have focused on the intention to commit DV, and the results were partially inconsistent. Prior studies of the utility of the TPB in violence-related contexts generally supports the model and the TPB explains approximately 16.4–44.8% of the variance in intentions [16,17,18,19]. Due to findings indicating that, on average, the TPB explains less than 50% of the variance in behavioral intentions, Ajzen (1991) proposed incorporating more variables into the model if the addition of those variables would enhance the TPB’s ability to predict behaviors and/or behavioral intentions.

External sociocultural factors also affect an individual’s behavioral intentions and/or behaviors, such as a history of family violence and gender stereotyping that have emerged as significant predictors of DV behavior [5,20,21,22]. A history of family violence refers to the overall domestic violence, father–child violence, mother–child violence, and the experience of witnessing parental violence [23]. A history of family violence is a risk factor that increases the likelihood of an individual becoming a victim or perpetrator of intimate partner violence [11,24,25]. Previous studies [5,21,22] reported that family violence experiences were closely related to the development of DV. For example, experiencing childhood maltreatment (e.g., father to child violence and mother to child violence) was a key factor of violence in a relationship; however, witnessing violence between one’s parents during childhood was not as consistent in predicting an individual’s subsequent involvement in a DV relationship [21,23].

Moreover, gender stereotyping, the product of the process of socialization, also affects DV behaviors [5,20]. Gender stereotype refers to the use of physical gender to assume the characteristics, roles, and functions of gender [26]. In traditional patrilineal societies, men have dominant authority, men are expected to be proactive, brave, etc.; women’s traits are gentle, submissive, etc. Gender stereotypes often lead to unequal treatment and resource allocation [26]. Moreover, gender influenced perception of domestic violence against women. Men in general saw domestic violence against women as a personal and family issue and did not recognize it as a serious social problem [27]. In addition, past studies have shown that gender stereotypes [5,20] and acceptance of DV [20] affect attitudes and behaviors of DV [26]. However, there is no research on gender stereotypes and DV in Taiwan. It is necessary to further explore the relationship between gender stereotypes and DV. We considered that expanding TPB may help explain the intentions to commit DV behaviors among university students as there may be other factors (i.e., a history of family violence or gender stereotyping) contributing to the intention to commit DV behaviors. Hence, we incorporated history of family violence and gender stereotyping into the TPB for our study.

In summary, it is critical to achieve a better understanding of the various correlates of university students’ intentions to commit DV behaviors (using a well-established TPB, history of family violence, and gender stereotyping) and then using this information to develop sensitive prevention programs. While various theoretical justifications for applying the extended TPB model to DV have been proposed, only a limited amount of empirical evidence that supports the application of the TPB, history of family violence, and gender stereotyping to the study of DV behaviors has been reported thus far. This study sought to address this existing gap in the literature on DV through an examination of the extended TPB model’s utility in exploring the intentions of university students to engage in DV.

As its first objective, this study sought to investigate the degree to which the components of the TPB are capable of interpreting the intentions of university students to behave violently toward their dating partners. Based on the suggestions of previous studies using the TPB in violence-related contexts generally supported TPB components to explain the behavioral intentions [16,17,18,19], the present study hypothesized that the various components of the TPB (that is, subjective norms, attitudes, and perceived behavioral control) could account, both individually and in combination with one another, for a significant amount of the variation in the intentions of university students to engage in DV behaviors.

The second objective of the present study was to utilize an expanded version of the TPB consisting of the original TPB model combined with the additional constructs of a history of family violence and gender stereotyping to investigate university students’ intentions to engage in DV. Since previous studies showed the DV was associated with age [9]. Moreover, gender was the predictor of cyber DV [28] and gender discrimination was 2.5 times more likely to report experiencing the highest frequency of DV [29]. Considering that human behaviors are often affected by age and gender, in order to explore the important factors affecting the intention of DV, age, and gender will be controlled in the statistical analysis. Relatedly, the study hypothesized that augmenting the related variables of the original TPB model with those additional constructs would, after controlling for background variables (age and gender), enhance the amount of the variation in the intentions of university students to engage in DV behaviors.

## 2. Materials and Methods

### 2.1. Study Design and Participants

The study adopted a cross-sectional and descriptive correlation study design. Participants were recruited from the three-step, stratified-random samples of the universities’ lists at the Ministry of Education for four regions in Taiwan: northern, southern, central, and eastern. This random sample of students was then stratified by, in order, school, then university, and then department. The desired sample used G power to calculate the number of samples, set α = 0.05, power = 0.8, number of independent variables = 11, effect size R^2^ = 0.375 [17], and the result of sample size was 99. Allowing for about a 20% withdrawal rate, the planned enrollment for the study was 120 subjects in each region. The targeted participants consisted of students in the first- to fourth-grade classes. Four schools among the total of 10 schools contacted in the four regions agreed to take part in the study. The criteria for inclusion were as follows: (1) at least 18 years old, (2) a daytime Taiwanese student, (3) able to speak Mandarin Chinese or Taiwanese, and (4) willing to participate in this study. We excluded students who belonged to an extension education center or who were married. The total actual sample sizes were 396–480. The pilot study was conducted in March 2018 with 50 subjects in Southern Taiwan, and the formal data were collected from April to May 2018. 

### 2.2. Measures

#### 2.2.1. Demographic and Related Variables

A brief self-reported questionnaire was utilized to collect the baseline demographic and dating-related data of each participant. The data included gender, age, year of study in university, region in Taiwan, religion, parents’ marital status, love experiences, and dating status.

#### 2.2.2. History of Family Violence

History of family violence was measured using a 3-item, self-measuring scale, which included history of violence by father, by mother, and witnessing of interparental violence. For example, “Have you ever suffered abusive or violent acts by your father, including physical or mental violence?”. Each item is scored from 0 (no) to 1 (yes). Summing the items’ ratings yields a score for history of family violence, with a higher score indicating more experiences of family violence. In the present study, Cronbach’s alpha of the overall history of family violence was 0.70.

#### 2.2.3. Gender Stereotyping

Gender stereotyping was measured using the gender stereotyping scale, which was developed by [30]. This scale is a 16-item, Likert-type scale, with 8 items representing the male gender stereotyping, and 8 items representing the female gender stereotyping. To evaluate male gender stereotyping and female gender stereotyping, the participants were asked what level of their agreement of characteristics, roles, and functions of gender. Example items include “When I see a boy crying in public, I feel that he is very cowardly” and “When the boy and the girlfriend get along, the girl has to play a submissive role”. Adequate reliability of the overall gender stereotyping scale has been demonstrated among central Taiwan college students (Cronbach’s alpha = 0.83). The scale utilizes a 5-point Likert scale for each item with responses ranging from strongly disagree (1) to strongly agree (5). Scoring was conducted by summing individual responses, with more male or female gender stereotyping being indicated by a higher score [30]. In the present study, Cronbach’s alpha for the overall gender stereotyping scale (sum of 16 items) was 0.88, female gender stereotyping was 0.85, and male gender stereotyping was 0.81.

#### 2.2.4. Theory of Planned Behavior Constructs: Attitudes, Subjective Norms, Perceived Behavioral Control, and Behavioral Intentions

The dating violence behavioral intention questionnaire (DVBIQ) was developed by [17] consistent with the TPB. The DVBIQ comprised a question about the student’s intention to commit DV and 25 items to measure the three constructs of TPB: attitudes (7 items), subjective norms (8 items), and perceived behavioral control (10 items). Each construct was comprised of direct measures and indirect (belief-based) measures. The general opinions and beliefs of each participant were assessed by direct measures and indirect measures (that is, measures of the specific underlying beliefs and outcome evaluations of the participant).

**Behavioral intention** to perpetrate DV was measured using one question: “If I have a dating partner, the possibility of my perpetrating dating violence against him/her in the following month is….”. Responses ranged from very unlikely (1) to very likely (7). A higher score reflected a greater intention to perpetrate DV.

**Attitude** refers to how an individual views DV, whether positively or negatively, as measured from his/her own perspective. Attitude was measured by evaluating the opinions of participants toward DV (that is, their direct attitudes), and their behavioral beliefs (that is, their beliefs that certain behaviors result in certain consequences) and outcome evaluations (that is, their evaluations regarding the positive and negative effects of such consequences). Participants answered 7 items on a 7-point scale. Example items include ‘‘In my opinion, physical, mental or sexual violence against a dating partner is…Strongly unacceptable (1) to strongly acceptable (7).”, “Physical, mental, or sexual violence against dating partner will make him/her obey me…Strongly disagree (1) to Strongly agree (7)”, and “Physical, mental, or sexual violence against a dating partner can make him/her obey me, I feel this is... Strongly bad (1) to Strongly good (7)”.

**Subjective norms (SN)** refer to the binding and norms of important others when individuals engage in DV. Subjective norms were measured by evaluating each participant’s perceptions of social pressure to perpetrate DV (with direct SN being measured using two items), normative beliefs (that is, the normative beliefs of particular referent groups), and motivation to comply (that is, comply with the normative beliefs of those groups). Participants answered 8 items on a 7-point scale. Example items include “Most of the people who influence me a lot (e.g., parents, friends, and teachers) hold an attitude that physical, mental, or sexual violence against a dating partner are….Strongly disagree (1) to Strongly agree (7).”, “Most of my teachers think that physical, mental or sexual violence against a dating partner is…Strongly unacceptable (1) to Strongly acceptable (7).”, and “Will I follow my teachers’ advice if I want to commit physical, mental or sexual violence against dating partner…Strongly unwilling to (1) to Strongly willing to (7).”

**Perceived behavioral control** refers to the ability of individual perception to control whether to perform violence against dating partners. Perceived behavioral control was measured by evaluating the participant’s perceptions of control and self-efficacy with regard to perpetrating DV (that is, the participant’s direct perceived behavioral control), controlling beliefs with regard to what enables the perpetration of DV, and perception regarding the strength of these factors. Participants answered 10 items on a 7-point scale. Example items include “To me, physical, mental, or sexual violence against a dating partner in the following month is…Strongly not easy (1) to Strongly easy (7).”, “If the dating partner hangs out with the person I worry about without informing me, that will provoke my physical, mental or sexual violence against her/him…Strongly possible(1) to Strongly impossible(7)”, and “If the dating partner hangs out with the person I worry about without informing me that will provoke my physical, mental or sexual violence against her/him. I feel this to be…Strongly possible(1) to Strongly impossible(7)”.

All measures were constructed from 7-point scales with every point appropriately labeled. The total score ranges for the subjective norms, attitudes, and perceived behavioral control subscales were 5–119, 5–161, and 7–217, respectively. A higher score reflected the individual having greater intention, favorable attitudes, greater perception of others’ expectations, or greater perceived behavioral control to perpetrate DV or accept DV as normal or deserved. The 26-item DVBIQ was previously validated for use with Taiwanese university students and showed good validity and reliability [17]. In the current study, the Cronbach’s alpha of the DVBIQ was 0.87, and that for the attitude, subjective norm, and perceived behavioral control subscales was 0.87, 0.75, and 0.90, separately.

### 2.3. Procedures and Ethical Considerations

This research project was approved by the Institutional Review Board of the University Ethics Committee (NCKU HREC-E-105-093-2). A pilot study (*n* = 50) was conducted at a university located in Southern Taiwan to test the reliability of questionnaires and problems during the research process, for example: time of finishing the questionnaires and wording problems. Prior to formal data collection, the corresponding author contacted school teachers and got support for conducting the study from each school. Teachers at each school contacted eligible participants for permission to conduct the study during a scheduled class. All students from the selected classes were queried for their permission to take part in the survey study.

The purposes, survey procedures, and potential risks and benefits of the study were explained to each participant by the corresponding author or a trained research assistant, as was the means by which the confidentiality of the participants would be protected. The voluntary and anonymous nature of the study was also emphasized by the author or the research assistant, as was each participant’s right to refuse participation in the study or to discontinue his or her participation at any time. After that, the self-reported paper questionnaires were distributed to consenting students. The study questionnaires were completed by the participants in approximately 10–15 min, and each participant was given a gift as a reward for his or her participation. Each participant’s consent to participate was regarded as being granted upon the participant’s return of the completed questionnaires.

### 2.4. Data Analysis

The analysis of the study data was performed using the statistical software IBM SPSS Statistics for Windows, version 22 (IBM Corp., Armonk, NY, USA), with the study sample being described using frequencies and other descriptive statistics. Cronbach’s alpha coefficient values were generated in order to evaluate the internal consistency of the questionnaire items in measuring the associated constructs, while a multiple regression analysis in which the three components of the TPB (that is, subjective norms, attitudes, and perceived behavioral control) were simultaneously entered as related factors of the behavioral intention to engage in DV was also conducted. Furthermore, a hierarchical multiple regression analysis that included the various control variables (that is, gender, age, religion, romantic experiences, dating status, and parents’ marital status) as the first step of the model was likewise conducted, with the second step of the model including the history of family violence and gender stereotypes and the third step of the model including the three components of the TPB. The dependent variable, meanwhile, consisted of the behavioral intention to engage in DV.

## 3. Results

In a classroom setting, 450 students voluntarily completed the self-measured questionnaires. Of the 450 surveys distributed, 365 (81.1%) questionnaires were valid after deleting incomplete questionnaires. The mean age of the participants was 20.32 years (SD = 1.41), with a range of 18–25 years. Further, 57.8% participants were female, 51.2% had religious beliefs, the numbers of participants in grades one to four were similar, and 29% participants’ schools were located in the southern district. Most participants reported their parents as married (83.6%). Of the participants, 59.7% had fallen in love with a dating partner, and 35.9% had a current dating partner. More details are provided in Table 1.

Table 2 shows that participants who had witnessed interparental violence (14.5%) saw more violence by mothers (7.1%) than by fathers (6.6%). Total score of family violence ranged from 0 to 3, with a mean score of 0.28 (SD = 0.69). Moreover, the mean score of male gender stereotyping (19.08) was higher than female gender stereotyping (14.02), and total gender stereotyping of participants was from 16 to 65, with a mean score of 33.10 (SD = 8.67). The mean scores of the three TPB components, that is, subjective norms, attitudes, and perceived behavioral control, were 12.36 (SD = 8.64), 7.53 (SD = 6.51), and 24.04 (SD = 28.18), respectively. The mean score for the intention to engage in DV was 1.22 (SD = 0.55). The mean scores of the four subscales of DVBIQ were in low score range for the measures used. In addition, Table 3 shows that gender, history of family violence, gender stereotyping, subjective norms, attitudes, and perceived behavioral control were all significantly associated with university students’ intentions to commit DV.

The first objective of this study was to determine the extent to which the various factors of the TPB are predictive of university students’ intent to engage in DV. In combination, the TPB components were significantly capable of predicting university students’ intentions to engage in DV, F(3, 161) = 48.66, *p* < 0.001, with the model accounting for 28.8% of the total variance (R^2^ = 0.288). Further investigation of the individual factors showed that both the attitudes towards behavior (β = 0.484, *p* < 0.001) and the subjective norms (β = 0.095, *p* = 0.037) of students were significant related factors of their intent to engage in DV (Table 4), results that suggest that the investigated university students who had positive attitudes regarding DV and the students who believed that their significant others would approve of their engaging in DV were more likely to have greater intentions to engage in DV behavior. Meanwhile, perceived behavioral control was not found to be a significant factor of the investigated students intentions to engage in DV.

In order to fulfill the second objective of this study, a hierarchical regression analysis examining the ability of the three TPB constructs to predict intentions to engage in DV was conducted, with a history of family violence, gender stereotyping, and background variables (age and gender) being controlled for. The results indicated that the model’s first step (age and gender) accounted for a significant amount of variance in university students’ intentions to commit DV, F(2, 363) = 3.071, *p* = 0.048, R^2^ = 0.017, Adj. R^2^ = 0.011. Step 2 of the model (addition of history of family violence and gender stereotyping) significantly accounted for an additional 4.7% (R^2^ change = 0.047) of the variance in university students’ behavioral intentions, F(4, 360) = 6.090, *p* = 0.000, R^2^ = 0.063 Adj. R^2^ = 0.053. However, the significant effect of gender was gone. Step 3 of the model (addition of TPB components) significantly accounted for an additional 24.1% (R^2^ change = 0.241) of the variance in university students’ behavioral intentions, F(7, 357) = 22.284, *p =* 0.000, R^2^ = 0.304, Adj. R^2^ = 0.290.

As shown in Table 5, the significant effects of gender, history of family violence and gender stereotyping are gone and only attitude towards the behavior remained as a significant related factor of university students’ intentions to commit DV. After controlling for age and gender, history of family violence, and gender stereotyping, we found that the three main variables of TPB (attitude, subjective norm, and perceived behavioral control) explained an additional 24.1%, and the total variance was 30.4%. Attitude toward DV is the most important related factor of intention to commit DV among university students in Taiwan. More specifically, university students with more positive attitudes towards DV revealed greater intentions to commit DV.

## 4. Discussion

In light of the substantial negative consequences of DV (which include physical injuries [31], the need for medical treatment of depression [5], suicidal ideation/behavior [32], and even homicide [33]), prevention programs addressing the specific developmental issues of university students are needed. However, only a few programs aimed at preventing DV among university students have been developed thus far in Taiwan. Moreover, while DV prevention programs designed specifically for university students are needed in Taiwan, such programs should be firmly grounded in evidence from relevant research. Relatedly, various behavioral theories, such as the theory of planned behavior (TPB) proposed by Ajzen, can be of assistance in seeking to determine what leads university students to engage in violent behaviors against their dating partners [16,17]. In fact, past research studies utilizing the TPB have demonstrated the model’s utility with respect to predicting variation in individual behavioral intentions to commit violent behaviors [16,17,18,19]. In the present study, meanwhile, an expanded version of the TPB that included history of family violence and gender stereotyping as additional constructs within the original TPB model was used to investigate the intentions of university students to engage in DV against their dating partners, even as the original TPB itself was also applied to determine its applicability to such behavior.

The study results indicated, as hypothesized, that the components of the TPB were significant related factors of the behavioral intentions of the investigated university students to engage in DV behaviors, accounting for 28.8% of the total variance in such intentions. This was broadly consistent with the results of prior studies that utilized the TPB to predict violence-related behavioral intentions, as those studies reported amounts of the total variance accounted for ranging from 16.4% to 44.8% [16,17,18,19]. Moreover, the results of the present study were also consistent with those of a study by Heirman and Walrave (2012) [18] regarding adolescents’ self-reported cyberbullying behavior indicating that the TPB components of attitudes and subjective norms were significant related factors of intentions to engage in such behavior. Relatedly, past research has indicated that, in developmental terms, both social pressure (that is, subjective norms) and the formation of attitudes when an individual is attending university may play a key role in determining the risky health-related behaviors of individual youths [34].

Meanwhile, the results of the present study indicated, surprisingly, that perceived behavioral control (that is, individuals’ perceived ability to control their own behaviors) was not a significant factor of the investigated university students’ intentions to engage in DV behaviors. Although this finding was not consistent with that of a past study by Hou et al. (2020) [17], who did find, when using the TPB, that perceived behavioral control was a significant related factor of the behavioral intentions of young people to engage in DV, it is possible that the frequency with which DV behaviors occur is so low (for example, some individuals may only have a conflict with a partner and engage in DV a few times within a single year) or that students regard DV behaviors as accidents that are not under their control (for example, they may regard them as occurring due to their partner’s problems) to such an extent that the investigated university students did not have confidence in their own ability to control DV behaviors. Nonetheless, there is still a need, in future research, to verify the role, if any, that perceived behavioral control plays in determining DV behaviors, because insights in this regard could be of value in the development of prevention programs. For example, if perceived behavioral control is ultimately identified as a significant related factor of the intention to engage in DV, then skills-based approaches could potentially be utilized to guide young people on how to prevent DV behaviors and/or on how to have a greater level of control when experiencing a conflict with a romantic partner who may pose a risk of committing DV.

The second objective of the present study was to advance the previous research on DV by utilizing an extended TPB framework to simultaneously examine the factors determining the intention to commit DV. The framework used included both history of family violence and gender stereotyping within the original TPB model as additional constructs, an approach that made it possible to identify similarities among the various contributing factors even under the constraints of a cross-sectional study design.

According to the hierarchical regression analysis results, the extended TPB accounted for 30.4% of the total variance in the investigated university students’ behavioral intentions to engage in DV, with the significant related factors of the students’ intentions being different in different steps of the model. Specifically, the significant related factors were gender, history of family violence and gender stereotyping, and attitudes in the first, second, and third steps, respectively. Meanwhile, when gender, history of family violence, and gender stereotyping were controlled for, only the TPB factor of attitudes was found to be a significantly related factor of the students’ behavioral intentions to engage in DV. These findings of the present study were consistent with those of previous studies [16,17,18,19] that also found the factor of attitudes to be the most important factor. Being a mediator with full mediation may be the reason why attitude was the only significant variable in the hierarchical regression. Specifically, family violence and gender stereotyping were significant in the second step and they became nonsignificant after attitude was entered into the regression model (i.e., the third step). Moreover, our correlation analyses (Table 3) showed the significant relationships between attitude and gender stereotyping/family violence. Therefore, it is likely that family violence and gender stereotyping associated with intention to engage in DV via attitude. In other words, family violence and gender stereotyping may shape an individual’s attitude toward DV; then, such an attitude subsequently associates with intention to engage in DV.

With a history of family violence and gender stereotyping being included in the model, it was found that the TPB component of attitudes towards DV behavior was, regardless of any other background variables, a significant related factor of the behavioral intentions of university students to engage in such behaviors. More specifically, the study results indicated that, among the investigated students, those who had relatively positive attitudes regarding DV behaviors indicated greater intentions to engage in such behaviors. Therefore, the results of the present study can be of considerable value for informing the development of DV prevention programs. For example, such programs could be designed to take aim at altering the attitudes of university students regarding DV behaviors, as opposed to focusing only on educating students regarding the potential risks of DV and identifying warning signs of DV behavior in partners of potential partners. Relatedly, given the relative lack of research on this subject, the results of the present study could be of assistance in taking major steps in the development of scientifically based DV prevention programs for university students.

Prevention programs aimed at addressing other health-related behaviors among young people, such as programs aimed at addressing social influences and norms, along with providing training in assertiveness to help students resisting peer pressure, are much more effective in terms of influencing factors beyond that of knowledge alone. As such, it is unsurprising that the results of the present study indicate that the effectiveness of youth DV prevention programs might be enhanced by addressing factors such as perceived social pressure and attitudes toward DV behavior. In fact, addressing these factors could potentially result in a reduction in behavioral intentions to engage in DV behaviors among young people, which could, in turn, decrease the levels of injuries resulting from DV in this population.

The present study had some limitations. First, while the theoretical framework used by the study to evaluate behavioral intentions to engage in DV is well established, the results of the study should nonetheless be interpreted with care because they are derived from only a single sample of university students in Taiwan and, as such, may not be generalizable to other countries and non-Asian samples. Second, the low mean and standard deviation for the dependent variable of behavioral intention (M = 1.22, SD = 0.55) was measured with a single-item scale. These values represent data reflecting subjects who are unlikely to have perpetrated abuse. Although the present sample had relatively low experience in perpetrated abuse, we believed that our findings still contribute to the literature. Specifically, the study of prevalence of perpetrated abuse among university students is shortage and this study is a nationwide survey that can reflect the situation and try to find relationships between variables. Moreover, many people do not think of their behavior as a kind of DV that may be the reason of low score of behavioral intension subscale. Therefore, it is hard to secure a large sample size with a relatively high score of DV to conduct the multiple regression analyses used in the present study. However, it is important for healthcare providers to understand the potential factors contributing to intention of committing DV. Thus, early intervention can be initiated to avoid the DV. With this regard, even our sample represents less on the population with violent behaviors, the associations found in the present study can provide some insightful information. In other words, our findings can be the initial point for healthcare providers and researchers to know the mechanisms. Future studies can subsequently, based on our findings, use the population with violent behaviors to test the mechanism. Meanwhile, healthcare providers may have some information to deal with this tough issue when future studies are collecting the population with violent behaviors. Moreover, we believe that the associations found between the variables will be somewhat comparable across different populations (i.e., population with violent behaviors and that without violent behaviors). Moreover, behavioral intention was measured with a single-item scale that does not measure interitem variance, and is not as reliable as multiple-item scales as one of our limitations, although the psychometric evidence of the “behavioral intention” has been supported by a recent article [17]. We encourage future researchers to modify the questionnaire we used for this study. Specifically, more items with plain language are needed to construct the “behavioral intention” and additional psychometric evidence is needed. Third, in this study, 59.7% of the participants had love experiences and only 35.9% of the participants had a current dating partner, which is a minority of the sample. Future studies should be conducted with the sample contained more college students who were in dating relationships that would be more reflective of current dating attitudes in actual relationships. Fourth, the instrument used to measure family violence did not ask for a concrete period of time or about different levels of intensity. Fifth, the present study did not include a prospective measure of DV behavior and, relatedly, that its cross-sectional design did not allow us to make any inferences regarding causality. Therefore, we suggest that future studies should be conducted with the aim of measuring actual DV behaviors in order to test the reported relationship between intentions and behaviors. Lastly, the ethnic minority population may have a different feature in the DV; for example, a study in Taiwan [35] that explored the experiences and attitudes of tribal involvement for indigenous intimate partner violence through the focus groups from four tribes. The result showed that the family elders’ meditation and involvement could not restrain the batterers’ violence as the traditional culture dissipated and the religious constraints weakened these days. It is worth noting that, the participants in the focus group of Paiwan were more supportive to the strategy of “tribal involvement” and also recognized the positive outcome of the “tribal involvement” when compared to Atayal, Amis, and Ruka. However, the present study did not request the participants to reveal their identity of ethnic minority. Therefore, we were unable to deeply investigate this important topic. Future studies are thus warranted to base one our findings to study the DV among ethnic minority population in Taiwan. However, regardless of the present study’s limitations, we believe that it provides sufficient information to provide subsequent studies with the ability to investigate the related factors of intentions to engage in DV behaviors while integrating history of family violence and gender stereotyping within the TPB model. Relatedly, the present study suggests interesting objectives and directions for future research.

## 5. Conclusions

The extended TPB explained 30.6% of the variance in university students’ intentions to commit DV in Taiwan. Using hierarchical regression analysis, the significant related factors of university students’ behavioral intentions were different at different steps, which were gender; a history of family violence and gender stereotyping; and attitudes in the first, second, and third step, respectively. After controlling background variables, a history of family violence, and gender stereotyping, only TPB attitude was a significant related factor of university students’ intentions to commit DV. It is hoped that these findings can be used in designing and implementing programs for the prevention of violent dating behaviors among university students in Taiwan.

## Figures and Tables

**Table 1:** Demographics and related variables of participants (*n* = 365).

Variable	Mean (SD)	*n* (%)
Gender		
Male		154 (42.2)
Female		211 (57.8)
Age (Range: 18–25 years old)	20.32 (1.41)	
Year of study in university		
First year		94 (25.8)
Second year		90 (24.7)
Third year		91 (24.9)
More than fourth year		90 (24.7)
Region in Taiwan		
Northern		86 (23.6)
Southern		106 (29.0)
Central		86 (23.6)
Eastern		87 (23.8)
Religious		
No		178 (48.8)
Yes		187 (51.2)
Parents’ marital status		
Married		305 (83.6)
Others (separate/widowed)		60 (16.4)
Love experiences		
No		147 (40.3)
Yes		218 (59.7)
Dating status		
No		234 (64.1)
Yes		131 (35.9)

**Table 2:** Related variables of present study (*n* = 365).

Variable	Mean (SD)	*n* (%)
Total family violence (Range: 0–3)	0.28 (0.69)	
History of violence by father		
No		341 (93.4)
Yes		24 (6.6)
History of violence by mother		
No		339 (92.9)
Yes		26 (7.1)
Witnessing of interparental violence		
No		312 (85.5)
Yes		53 (14.5)
Total gender stereotyping (Range: 16–65)	33.10 (8.67)	
Male gender stereotyping (Range: 8–39)	19.08 (5.13)	
Female gender stereotyping (Range: 8–32)	14.02 (4.67)	
Attitude toward dating violence (Range: 5–53)	7.53 (6.51)	
Subjective norm (Range: 5–48)	12.36 (8.64)	
Perceived behavioral control (Range: 6–198)	24.04 (28.18)	
Intention to commit dating violence (Range: 1–4)	1.22 (0.55)	

**Table 3:** Correlation of related variables in present study (*n*= 365).

	1	2	3	4	5	6	7	8	8-1	8-2	9	10	11	12
1 Gender	-													
2 Age	0.039	-												
3 Religious	−0.043	0.080	-											
4 Love experience	−0.102	0.163 **	−0.019	-										
5 Currently dating	−0.084	0.188 **	0.044	0.568 **	-									
6 Parent’s ms	−0.010	0.057	−0.033	−0.108 *	−0.100	-								
7 Family violence	0.004	0.028	0.074	0.012	−0.008	−0.162 **	-							
8 Total GS	0.292 **	−0.055	−0.043	0.044	0.089	0.119 *	−0.025	-						
8-1 mGS	0.120 *	−0.031	−0.007	0.087	0.102	0.123 *	0.025	0.896 **	-					
8-2 fGS	0.410 **	−0.067	−0.072	−0.015	0.054	0.086	−0.073	0.873 **	0.566 **	-				
9 AB	0.078	0.033	−0.018	−0.003	−0.040	−0.012	0.157 **	0.120 *	0.095	0.119 *	-			
10 SN	0.193 **	−0.029	−0.032	0.042	0.066	−0.021	0.026	0.304 **	0.226 **	0.315 **	0.181 **	-		
11 PBC	−0.019	0.015	0.012	0.029	0.042	−0.027	0.095	0.160 **	0.149 **	0.133 *	0.428 **	0.202 **	-	
12 BI	0.123 *	0.043	−0.020	0.002	−0.039	−0.011	0.155 **	0.174 **	0.145 **	0.164 **	0.525 **	0.194 **	0.281 **	-

Note. Parent’s ms = parent’s marital status; Total GS = total gender stereotyping; mGS = male gender stereotyping; fGS = female gender stereotyping; AB = attitude toward dating violence; SN = subjective norm; PBC = perceived behavioral control; BI = behavioral intention of dating violence; ** *p* < 0.01; * *p* < 0.05.

**Table 4:** Multiple regression analysis for factors associated with the intention to commit dating violence from TPB components.

Variable	B	Std. Error	β	t	*p*
Attitude	0.041	0.004	0.484	9.083	**0.000**
Subjective norm	0.006	0.003	0.095	2.094	**0.037**
Perceived behavioral control	0.001	0.001	0.054	1.088	2.777
F	48.66				
Adjusted R^2^	0.282				
Total R^2^	0.288				

**Table 5:** Hierarchical regression analysis results regarding the ability of the TPB components to predict the intention to engage in dating violence from when controlling for various background variables (*n* = 365).

Variable	ΔR^2^	Adjusted R^2^	B	Std. Error	β	t	*p*
Step 1	0.017	0.011					
Age			0.015	0.020	0.038	0.726	0.468
Gender			0.136	0.058	0.122	2.339	**0.020**
Step 2	0.047	0.053					
Age			0.017	0.020	0.044	0.860	0.390
Gender			0.083	0.060	0.075	1.399	0.163
Gender			0.086	0.059	0.077	1.451	0.148
Family violence			0.127	0.041	0.158	3.093	**0.002**
Gender stereotyping			0.010	0.003	0.159	2.973	**0.003**
Step 3	0.241	0.290					
Age			0.011	0.017	0.029	0.649	0.517
Gender			0.056	0.052	0.051	1.082	0.280
Family violence			0.062	0.036	0.078	1.739	0.083
Gender stereotyping			0.062	0.036	0.077	1.724	0.086
Attitude			0.039	0.004	0.466	9.369	**0.000**
Subjective norm			0.004	0.003	0.065	1.362	0.174
Perceived behavioral control			0.001	0.001	0.049	0.976	0.330
F	22.284						
Total R^2^	0.304						

## Data Availability

The data presented in this study are available on request from the corresponding author.
